# Performances Evaluation of a Low-Cost Platform for High-Resolution Plant Phenotyping

**DOI:** 10.3390/s20113150

**Published:** 2020-06-02

**Authors:** Riccardo Rossi, Claudio Leolini, Sergi Costafreda-Aumedes, Luisa Leolini, Marco Bindi, Alessandro Zaldei, Marco Moriondo

**Affiliations:** 1 Department of Agriculture, Food, Environment and Forestry (DAGRI), University of Florence, Piazzale delle Cascine 18, 50144 Florence, Italy; sergi.costafredaaumedes@unifi.it (S.C.-A.); luisa.leolini@unifi.it (L.L.); marco.bindi@unifi.it (M.B.); 2 Independent Researcher, via dei Tigli 37, 50041 Florence, Italy; claudio.leolini@gmail.com; 3 CNR-IBE, via Madonna del Piano 10, 50019 Florence, Italy; alessandro.zaldei@ibe.cnr.it (A.Z.); marco.moriondo@cnr.it (M.M.)

**Keywords:** 3D phenotyping, low-cost platform, plant imaging, structure for motion

## Abstract

This study aims to test the performances of a low-cost and automatic phenotyping platform, consisting of a Red-Green-Blue (RGB) commercial camera scanning objects on rotating plates and the reconstruction of main plant phenotypic traits via the structure for motion approach (SfM). The precision of this platform was tested in relation to three-dimensional (3D) models generated from images of potted maize, tomato and olive tree, acquired at a different frequency (steps of 4°, 8° and 12°) and quality (4.88, 6.52 and 9.77 µm/pixel). Plant and organs heights, angles and areas were extracted from the 3D models generated for each combination of these factors. Coefficient of determination (R^2^), relative Root Mean Square Error (rRMSE) and Akaike Information Criterion (AIC) were used as goodness-of-fit indexes to compare the simulated to the observed data. The results indicated that while the best performances in reproducing plant traits were obtained using 90 images at 4.88 µm/pixel (R^2^ = 0.81, rRMSE = 9.49% and AIC = 35.78), this corresponded to an unviable processing time (from 2.46 h to 28.25 h for herbaceous plants and olive trees, respectively). Conversely, 30 images at 4.88 µm/pixel resulted in a good compromise between a reliable reconstruction of considered traits (R^2^ = 0.72, rRMSE = 11.92% and AIC = 42.59) and processing time (from 0.50 h to 2.05 h for herbaceous plants and olive trees, respectively). In any case, the results pointed out that this input combination may vary based on the trait under analysis, which can be more or less demanding in terms of input images and time according to the complexity of its shape (R^2^ = 0.83, rRSME = 10.15% and AIC = 38.78). These findings highlight the reliability of the developed low-cost platform for plant phenotyping, further indicating the best combination of factors to speed up the acquisition and elaboration process, at the same time minimizing the bias between observed and simulated data.

## 1. Introduction

Plant phenotyping is recognized as one of the most relevant research fields addressing crop genetic improvement [1] by providing significant advances in agronomic and breeding programs [2]. Indeed, plant phenotypes are the expression of similar genes [3], which, coupled with exogenous factors, contribute to modifying the morphological structure from the early stages of growth [4]. The evaluation of this interaction through the analysis of particular morphological traits of stem, petioles/branches and leaves allows the most stress-tolerant genotypes to be selected, also taking into account the expected impact of climate change [5]. For such a purpose, in recent years, different approaches were developed for efficient and accurate quantitative measurements of phenotypic traits related to plant growth and architecture, including size and shape [6].

The first studies on plant phenotype were conducted through destructive and time-consuming methods [7], which in many cases resulted in inefficient and inaccurate measurements of morphometric traits [8]. The technology improvement in the last decades has allowed the introduction of new non-invasive tools, ensuring a rapid and faithful structural parameters’ extraction [9]. In this context, crop phenotyping platforms are considered a valid solution [10]. Phenotyping platform-based approaches enable high-throughput, automated and simultaneous multiple-plant screening, resulting in saving processing time and improved accuracy in the analysis of plant traits [11]. 

Automated platforms have been designed mainly for plant phenotyping in growth chambers or greenhouses and combine robotics, remote sensors and data analysis systems [12]. The most efficient hardware setup requires control devices mounted on the platform to automatically move sensors over the plants [13]. Several sensor-to-plant commercial phenotyping systems have been successfully used for specific applications, such as analysis of plants’ growth [14], sensitivity to water stress [15] and response to light alterations [16]. However, the complex structure of these platforms necessitates specific layouts of growth chambers or greenhouses [17], which are unsuitable for a widespread use by researchers and breeders [18]. 

The high cost of commercial platforms has driven stakeholders to develop simpler facilities equipped with a range of sensors, including RGB digital cameras [19], light detection and ranging (LiDAR; [10]) and depth scanners [20]. These platforms ensure faithful plant three-dimensional (3D) modelling for morphological parameters’ measurement, combining non-invasive imaging and image analysis [21]. Nevertheless, many devices may not be initialized for affordable phenotyping and breeding programs due to sensors’ cost-efficiency and spatial and temporal resolution [22], and to fill this gap, low-cost imaging solutions are gaining interest [23,24].

Among these techniques, structure for motion (SfM) represents a widely-used approach for plant phenotyping from both proximal and remote platforms [25,26,27,28]. SfM is based on the overlap of a set of two-dimensional (2D) images acquired by digital camera(s) from multiple viewing angles for 3D-object reconstruction [29]. Unlike conventional photogrammetric techniques, SfM automatically estimates the geometry of the scene and camera parameters [30], requiring less user supervision and expertise for plant modelling [31].

A camera-based SfM approach has been used for a low-cost and detailed reconstruction of herbaceous and tree crops, such as soybean [32], paprika [9] and olive tree [26]. For instance, Liu et al. [33] built a 3D representation of a manually-rotated single rosette plant, acquiring 1000 low-resolution images (7.96 megapixels), which is too labor-intensive for a multi-plant application. Similarly, Santos and de Oliveira [34] captured 143 and 77 images at 9.98 megapixels resolution for modelling basil and ixora specimens, stating that their method is not suitable for automatic plant digitizing due to time-consuming image-processing and occlusion problems, especially in too-dense canopies. 

Indeed, although results were satisfactory, no studies have provided references for optimizing the reconstruction process in terms of input and time requirements, and this represents a bottleneck in the prompt and automatic acquisition of plant phenotypic traits. As previously reported by Torres-Sánchez et al. [35], processing time depends on the quantity and resolution of images used in models’ generation. Zhou et al. [36] developed a SfM-based budget phenotyping platform to find a balance between processing time and accuracy in measuring geometric dimensions of 44 soybean plants in a greenhouse. They showed that plant height can be faithfully reconstructed by spending approximately 5.5 h to process 156 images with 19.91 megapixels resolution. However, this input combination is applicable solely on one species and for the extraction of a single morphological parameter. 

To our knowledge, no other study has attempted to establish the proper balance between number of images and their quality for an efficient and accurate measurement of individual structural parameters for species with different canopy structure. In this context, a comprehensive analysis based on the effectiveness and reproducibility of the image-based SfM technique is needed [37] for the reconstruction of high-precision models and the extraction of complex morphological parameters [9,25].

To meet the needs stated in this premise, the aim of this paper is two-fold: firstly, developing a low-cost and automated platform equipped with a conventional RGB camera for 3D plant reconstruction using an SfM approach, and as a second step, we tested the reliability of this platform in the faithful reproduction of main plant phenotypic traits (plant and branches height, stem diameter, petioles/branches inclination and single-leaf inclination and area) in relation to the frequency and quality of acquired images. To get the widest possible overview of the performance of this platform, this test phase was carried out on species with completely different canopy architectures, namely maize (*Zea mais* L.), tomato (*Lycopersicum esculentum* L.) and olive tree (*Olea europaea* L.). The methodology was evaluated considering the best compromise between image frequency and quality of acquisition that at the same time permits us to improve processing times and obtain reliable morphometric data.

## 2. Materials and Methods

### 2.1. Phenotyping Platform Setup

**Figure 1:**
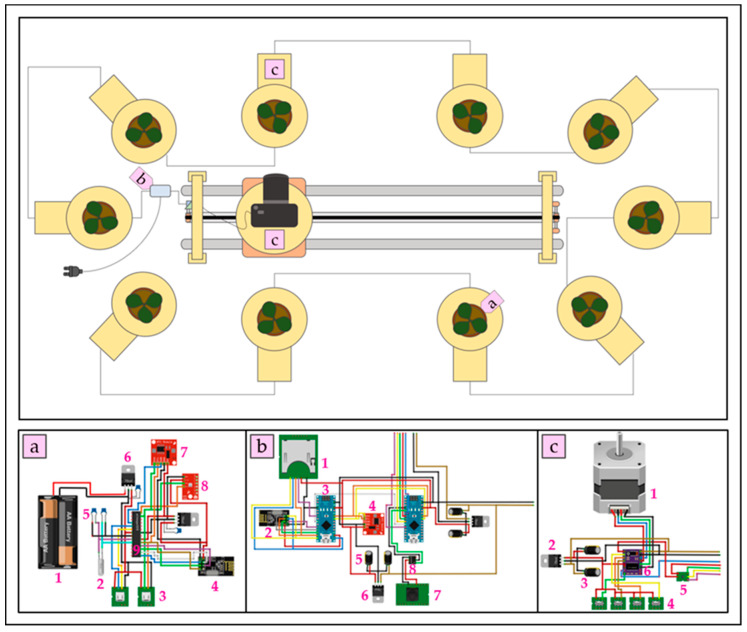
Schematic representation of the low-cost and automatic phenotyping platform. The platform consists of the following electronical components: (**a**) data acquisition module with (a1) 2 AA batteries, (a2) a quartz crystal oscillator (16 MHz), (a3) 2 micro-USB connectors for weight and humidity sensors, (a4) a NRF24L01 transceiver module (2.4 GHz; Nordic Semiconductor^®^, Trondheim, Norway), (a5) 4 capacitors (two 22 pF, one 470 pF and one 1 uF), (a6) 2 voltage regulators 78xxl (1 mic5205-3.3y and 1 mic5205-5.0y; Micrel^®^, San Jose, CA, USA), (a7) a BME280 temperature, humidity and pressure sensor (Digi-Key^®^, Thief River Falls, MN, USA), (a8) a RTC, and (a9) a MCU ATmega328P_PU. (**b**) Data logger module with (b1) an SD card reader, (b2) a NRF24L01 transceiver module (2.4 GHz), (b3) 2 Arduino Nano V3.0, (b4) a RTC, (b5) 4 470 pF capacitors, (b6) 2 voltage regulators 78xxl (mic5205-5.0y), (b7) photographic camera module, and (b8) a 4N35 optocoupler (Mouser Electronics^®^, Solsona, Barcelona, Spain). (**c**) Engine control module with (c1) stepper motor (400 steps), (c2) a voltage regulator 78xxl (mic5205-5.0y), (c3) 2 470 pF capacitors, (c4) 4 switches (1 for direction and 3 for micro-steps control), (c5) engine enable unit based on Boolean logic, and (c6) driver DRV8825 (Texas Instruments Incorporated^®^, Dallas, TX, USA).

The platform (Figure 1) is basically composed of a common RGB camera installed on a sliding system consisting of two 1 m long aluminum tubes, which allows the camera to position itself in front of the target/plant to be scanned. The plants are placed on rotating plates (Ø 15 cm) equipped with soil moisture and weight sensors for real time monitoring of plant transpiration rate. The camera is mounted on a support equipped with thrust-ball bearings that enable camera rotation on its vertical axis so as to allow the positioning of the rotating plates on both sides of the track. This simplified setup made the developed platform space-saving and flexible in adapting to the majority of growth chambers or greenhouses. 

All hardware components are coordinated by low-power MicroController Units (MCUs; 2 Arduino Nano V3.0 based on ATmega328P_PU and standalone ATmega328P_PUs; https://www.arduino.cc). At the appointed time, a MCU (Arduino^®^ Nano V3.0; Atmel Corporation^®^, San Jose, CA, USA) equipped with a Real-Time Clock (RTC, DS1307; Maxim Integrated^®^, San Jose, CA, USA) module sends an impulse to the DRV8825 stepper motor MCU (Arduino^®^ Nano V3.0; Texas Instruments Incorporated^®^, Dallas, TX, USA) positioned on the guidance system and on the plates. The mechanical automatism allows camera shots to be synchronized with the movement of the stand and guarantees a single-plant whole-shape scanning. We developed an algorithm in Arduino^®^ Integrated Development Environment (IDE) for programming the path of the camera and the frequency and speed of plates’ rotation.

Once the series of images is captured, the camera moves towards the next target and begins a new 360° acquisition. At the end of the cycle, it returns to the initial position and waits for a new signal from the RTC module. At the same time, a MCU (standalone ATmega328P_PU; Atmel Corporation^®^, San Jose, CA, USA) inserted on each pot sends the soil moisture and plant weight via Wi-Fi by low-cost sensors (VH400, Vegetronix^®^ and load cells interfaced with HX711 Balance Modules, respectively; Avia Semiconductor Co.^®^, Xiamen, China) to the RTC-equipped microcontroller for saving on a Secure Digital (SD) card. 

The components we used to assemble the platform were readily available on the online market or alternatively in local stores with low prices. Accordingly, the total cost of the system, including sliding track, ten rotating plates, engine control modules (Supplementary Table S1a) and data logger (Supplementary Table S1b) was about $800. Also considering the data acquisition modules for single-plant monitoring (not used in this study), the price increased by about $500 (Supplementary Table S1c). In both cases, the platform can be considered a low-cost phenotyping system comparing to commercial and private solutions. This platform was tested in an automated 360° scanning of each target-sample based on the SfM approach.

### 2.2. Experimental Setup and Observed Data

The experiment of plant phenotyping reconstruction was conducted on 12 potted plants of three different species (*Zea mays* L., *Lycopersicum esculentum* L. and *Olea europea* L.). The species were selected according to their high-agro-economical interest and morphological structure, typical of herbaceous and tree crops. 

Maize and tomato seedlings were sown in single pots (13 × 13 × 12 cm) and located in an environmentally controlled growth chamber for over 3 weeks. In order to replicate optimal growth conditions, the chamber’s internal air temperature was 27 °C for 12 h/d in lighting conditions (Osram Fluora L 36W/77^®^ fluorescence lamps at 1400 Lumen; OSRAM Licht AG^®^, Munich, Germany) and 18 °C for the remaining time in dark conditions, in order to simulate the diurnal sunlight cycle. Instead, 2-year-old olive trees were planted in single pots (10 × 10 × 25 cm) and grown in a heated greenhouse (maximum and minimum temperature of 20 °C and 10 °C, respectively) during the cold season, and outdoors in natural environmental conditions for the remaining time. All the 12 plants were fully irrigated during the growing season. The measurements were conducted 21 d after sowing (DAS) for maize, 24 for tomato and 810 and 811 for olive tree, under indoor controlled illumination. In particular, uniform lighting of the single plants was guaranteed by two neon photography lamps at a color temperature of 5400 K, following Biasi et al. [38]. In order to avoid similarities in color between the background and foreground samples, a vertical red panel was placed behind the scene. Four markers with a center point radius of 10 mm were applied to each pot and further used for generating and rescaling the point cloud. A Nikon^®^ D810 36.3 megapixel (Nikon Corporation^®^, Minato, Tokyo, Japan) digital camera sensitive to the visible range (400–700 nm) of the electromagnetic spectrum, equipped with a Nikon^®^ AF-S DX NIKKOR 35 mm f/1.8G (Nikon Corporation^®^, Minato, Tokyo, Japan) lens was mounted on the platform. In order to avoid radiometric image distortion [39] and to increase reconstruction accuracy [40], the camera was placed on the sliding system with a decreasing inclination angle around 15 degrees with respect to the plant and set in manual-exposure mode. 

For each target, a set of 90 images was acquired at a regular step of 4° with three different quality levels in terms of pixel resolution: high (4.88 µm/pixel; H), medium (6.52 µm/pixel; M) and low (9.77 µm/pixel; L).

**Figure 2:**
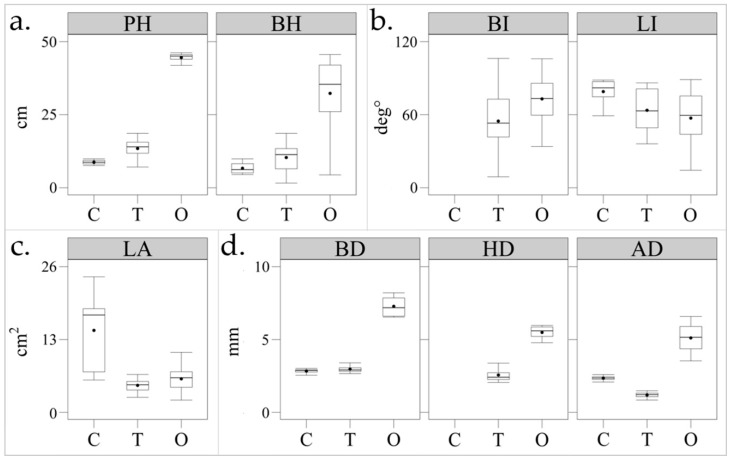
Observed morphological values of: (**a**) plant height (*PH*) and branches heights (*BH*), (**b**) leaves (*LI*) and branches inclination (*BI*), (**c**) single-leaf area (*LA*) and (**d**) basal (*BD*), half-plant (*HD*) and apical (*AD*) stem diameter for maize (*C*), tomato (*T*) plants and olive trees (*O*) considered in the study.

Subsequently, observed data of plant height (*PH*), petioles/branches height (*BH*), basal, half-plant and apical stem diameter (*BD*, *HD*, *AD*), petioles/branches inclination angle (*BI*) and single leaf inclination (*LI*) and leaf area (*LA*) of a specific subset of leaves were measured (Figure 2).

Particularly, plant heights were measured from the lowest point of the main stem to the top of the plant (*PH*) and to the insertion of each branch (*BH*) using a tape measure with markings every millimeter. Presupposing that the seedlings’ stem had a circular cross-section, *BD*, *HD* and *AD* were obtained with a single measurement at each of the three heights using a digital caliper with a submillimeter precision (0.01 mm). Over each plant, sampled leaves were labelled by a marker placed on the upper side of the leaf and a smartphone-camera application (Camera Angle^®^; https://y-dsgn.com/angle.html) was used to obtain levelled images that were used to measure *BI* and *LI* using an image editor. Leaves were then removed from the plants, placed on a gridded plane and photographed orthogonally from above for calculating *LA* (see Section 2.3).

From the entire dataset, three groups of digital images were selected (90, 45 and 30 photos), considering 30 as the minimum number of images for a proper reconstruction [40,41].

### 2.3. Image Processing

**Figure 3:**
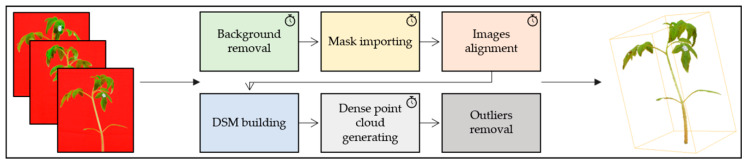
Workflow of image processing with specific timed steps.

The images were processed on an Intel^®^ Xeon^®^ Central Processing Unit (CPU) E3-1270 v6 3.80 GHz workstation (Santa Clara, CA, USA) with 32 GB RAM according to the following procedure (Figure 3). Firstly, the red background panel was automatically removed using a supervised color-thresholding approach in MATLAB^®^ environment (ver. R2018b; The MathWorks Inc. ^®^, Natick, MA, USA). Masks obtained were further used to generate a dense 3D-point cloud of the plant with Agisoft PhotoScan Professional^®^ (ver. 1.4.1; Agisoft LLC^®^, St. Petersburg, Russia). This software allowed us to automatically refine the camera position and orientation for each photo loaded using the markers placed on each pot as ground control points (GCPs) and to build a digital surface model (DSM) by searching for common points in the scene. After obtaining the sparse cloud, a solid 3D model (xyz) was created by setting high-quality reconstruction, in order to obtain a detailed plant geometry representation in a reasonable time [42]. The dense point cloud was scaled using the markers as references. Finally, outlier points were sorted out using an inbuilt statistical filter. The amount of time required for each main step was recorded for every sample. 

The open source image processing software ImageJ^®^ (http://rsbweb.nih.gov/ij/) was adopted to detect the angles of petioles/branches (*BI*) and leaves (*LI*) and to calculate the area of the leaves (*LA*). *BI* were obtained considering the vertical angle between a single petiole/branch and stem [32]. We measured *LI* as the angle between the leaves’ surface normal and the zenith, following the methodology used in previous studies [26,43,44]. *LA* was calculated by color-thresholding the gridded background and setting the surface included within the leaf boundaries [26,45,46]. 

### 2.4. Architecture Segmentation

Plants 3D reconstructions were visualized and manually segmented into principal stem, petioles/branches and single leaves using CloudCompare^®^ (http://www.cloudcompare.org/). All the xyz points that defined each plant organ were manually digitized and classified/segmented individually. A MATLAB^®^ semi-automatic algorithm was used for extracting plant morphology.

Following Wu et al. [47], $\hat{PH}$ and $\hat{BH}$ were simulated considering the straight-line distance in the Euclidean space ($\hat{H})$ between a common xyz point (*p1*) and another two xyz points (*p3* and *p4*, which are the highest point of the main stem and lowest point of each petiole/branch dense cloud respectively, Equation (1)).

(1) $$\hat{H}=\sqrt{{\left(x_{pi}-x_{p1}\right)}^{2}+{\left(y_{pi}-y_{p1}\right)}^{2}+{\left(z_{pi}-z_{p1}\right)}^{2}}$$

where *p1* is the lowest point of the dense cloud, *pi = p3* or *p4*, $x_{pi}\;\;\;$is the *x*-coordinate of the point *i*, $y_{pi}\;\;\;$is the *y*-coordinate of the point *i* and $z_{pi}\;\;\;$is the *z*-coordinate of the point *i*.

$\hat{BD},\;\;\;\hat{HD}\;\mathrm{and}\;\hat{AD}$ were measured considering the methodology presented by Gélard et al. [48]. Firstly, three different rings with a fixed radius (depending on the plant species) and characterized by *p1*, stem midpoint (*p2*) and *p3* as centroids respectively, were created. Secondly, all the points of the stem point cloud contained within each ring were selected, and the stem diameter at the three heights ($\hat{D}$) was extracted (Equation (2)).

(2) $$\hat{D}=min\left(\frac{rangeX}{2},\frac{rangeY}{2}\right)$$

where *rangeX* and *rangeY* are the differences between the highest and lowest *x*- and y-coordinate values respectively, of all the points contained within each ring.

$\hat{BI}$ was estimated by the angle between the hypothetical single petiole/branch plane and the zenith. For each set of coordinates representing a leaf, the best fitting-plane to 3D data was automatically detected and the angle between the vector normal to that plane and the leaf surface zenith was obtained ($\hat{LI}$). Subsequently, a boundary that enveloped all the single-segmented leaf points was created using a convex hull approach and $\hat{LA}$ was calculated, also including any internal voids.

### 2.5. Statistical Analysis

The coefficient of determination (R^2^, Equation (3), [49]), the relative Root Mean Squared Error (rRMSE, Equation (4), [50]) and the Akaike Information Criterion (AIC, Equation (5), [51]) were used as goodness-of-fit indexes to compare the simulated to the observed morphological measurements. According to previous studies [52,53], model accuracy is considered excellent when rRMSE ≤ 10%, good if 10% < rRMSE ≤ 20%, fair if 20% < rRMSE ≤ 30% and poor if rRMSE > 30%. A weighting adjustment (*Sw*, Equation (6)) was applied to the above-mentioned statistical indexes in order to compensate for missing values in the 3D reconstruction.

(3) $$R^{2}=\frac{\sum_{i=1}^{n}{\left(O_{i}-P_{i}\right)}^{2}}{\sum_{i=1}^{n}{\left(O_{i}-\bar{O}\right)}^{2}}$$

(4) $$\;\mathrm{rRMSE}=\frac{{\left[\sum_{i=1}^{n}\frac{{\left(P_{i}-O_{i}\right)}^{2}}{n}\right]}^{0.5}}{\bar{O}}\times 100\;$$

(5) $$\;\mathrm{AIC}=2k-2\ln\left(\hat{L}\right)\;$$

(6) $$\;\mathrm{Sw}=S\;\left[1-\left(\frac{\sum_{i=1}^{n}mv}{n}\right)\right]\;$$

where $O_{i}\;\;\;$is the observed value *i*, $\bar{O}$ is the average of the observed values, $P_{i}\;\;\;$is the simulated value *i*, *n* is the number of observations, *k* is number of estimated parameters in the model, $\hat{L}$ is the maximum value of the likelihood function for the model, *S* is the statistical index considered and *mv* is the number of missing values. 

## 3. Results

### 3.1. Time Processing

**Figure 4:**
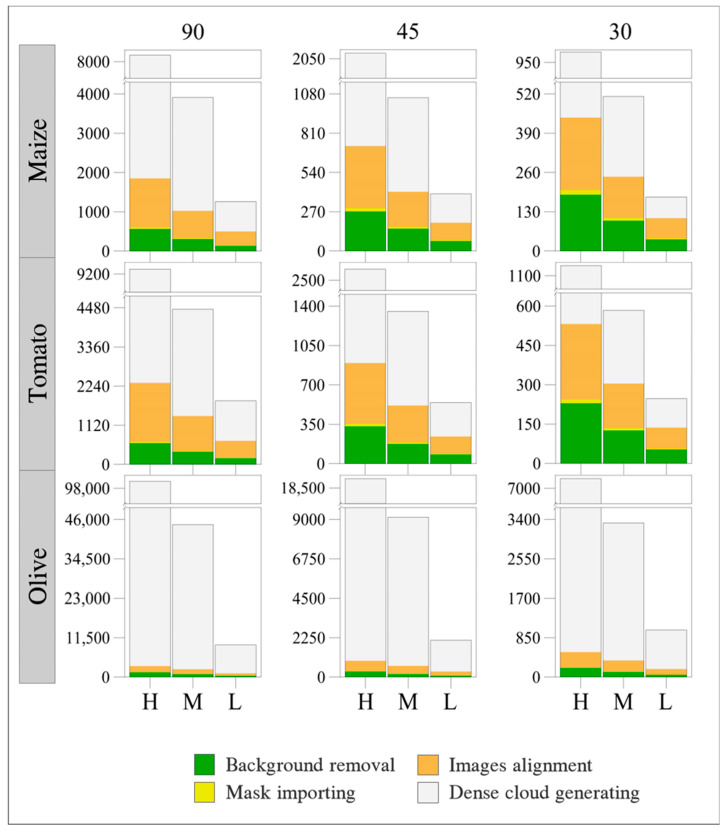
Average time, in seconds (sec), required for each main step (background removal, mask importing, images alignment and dense cloud generating) of image processing for the three-dimensional (3D) reconstruction of maize, tomato and olive-tree plants considering every combination of photo quantity (90, 45, 30) and quality (H, M, L).

The 3D SfM-based reconstruction procedure of herbaceous crops (maize and tomato) resulted consistently as more time-saving compared to olive trees (Figure 4). Based on the average time at 30 images per variety, the processing time increased on average from 2.2 to 7.7 times for maize and tomato and from 2.5 to 12.2 for olive tree at 45 and 90 images, respectively. Equally, the average time required for image processing at 9.77 µm/pixel increased from 2.0 to 5.4 times for herbaceous crops, and from 2.2 to 9.1 for olive tree, at 6.52 and 4.88 µm/pixel spatial resolutions. Among the different main steps of image processing (background removal, mask importing, images alignment and dense cloud generating), the generation of a dense point cloud was the most time-consuming for each species. It took on average 59.6% (maize and tomato) and 90.9% (olive tree) of the total amount of time and was followed by the images’ alignment (24.8% and 6.2%), background removal (14.2% and 2.6%) and masks’ importing (1.2% and 0.2%). The remaining time (<0.1%) was attributable to outlier points removal. 

### 3.2. Morphometric Traits Extraction

**Figure 5:**
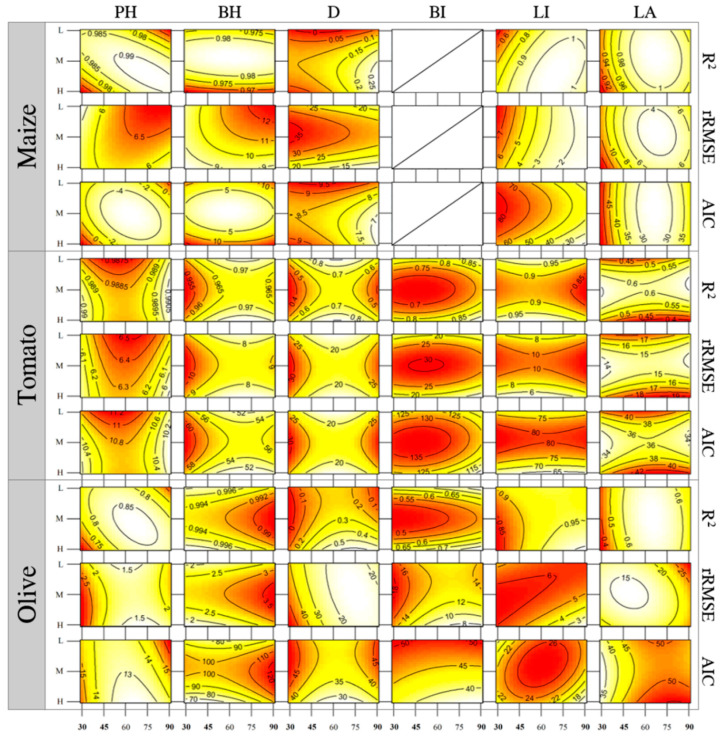
Response surface methodologies (RSMs) of maize (C), tomato (T) and olive-tree (O) plant heights (*PH* and *BH*; cm), merged basal, half-plant and apical stem diameter (D; mm), petioles/branches inclination (*BI*; deg), leaves’ inclination (*LI*; deg) and leaf area (*LA*; cm^2^) predictors (*R*^2^, rRMSE and AIC) related to image quantity (30, 45, 90; on the axis of ordinates) and quality (L, M, H; on the axis of abscissas) changes.

The overall results indicated, as expected, that the best performances in reproducing the main plant morphological traits of all varieties was obtained by combining the lowest rotating step (4°) for the highest quality of images (4.88 µm/pixel; Figure 5). In general, this combination of factors guaranteed a high agreement (R^2^ = 0.81) and excellent accuracy (rRMSE = 9.49%) at estimating most plant parameters (Table 1), except the grouping of stem diameters at the three different heights (*D*) and *LA* (only for tomato and olive tree). 

#### 3.2.1. Plant Heights

Simulated *PH* and *BH* showed strong agreement in maize (R^2^ ≥ 0.97), tomato (R^2^ ≥ 0.95) and olive tree (R^2^ ≥ 0.70), always with a good error index (rRMSE ≤ 12.87%), irrespective of input quantity and quality. In particular, the accuracy in *PH* estimation of maize (rRMSE ≤ 7.05%), tomato (rRMSE ≤ 6.64%) and olive tree (rRMSE ≤ 3.33%) was higher than in *BH* (rRMSE ≤ 12.87%, 12.74% and 5.20%, respectively). By considering the AIC values, the fits for *PH* were also better than those in *BH*. For both parameters, the reconstruction performances were not significantly improved when passing from the lowest to highest input requirements (Figure 5) and the combination of 30 low-resolution images may be considered the most effective in terms of time and accuracy for evaluating *PH* and *BH* in maize (R^2^ = 0.97, rRMSE = 5.15% and AIC = 2.36; R^2^ = 0.98, rRMSE = 8.41% and AIC = 3.49), tomato (R^2^ = 0.99, rRMSE = 6.23% and AIC = 10.65; R^2^ = 0.93, rRMSE = 12.74% and AIC = 69.95) and olive tree (R^2^ = 0.83, rRMSE = 3.33% and AIC = 14.19; R^2^ = 0.99, rRMSE = 2.07% and AIC = 82.22). 

#### 3.2.2. Stem Diameters

The estimation of *BD*, *HD* and *AD* (merged as *D* in Figure 5) evidenced the highest average performances for tomato (*R*^2^ ≤ 0.75, 19.73% ≤ rRMSE ≤ 35.62% and 19.11 ≤ AIC ≤ 34.10) followed by maize (R^2^ ≤ 0.23, 11.80% ≤ rRMSE ≤ 37.18% and 6.03 ≤ AIC ≤ 9.39) and olive tree (R^2^ ≤ 0.56, 16.39% ≤ rRMSE ≤ 54.29% and 24.04 ≤ AIC ≤ 45.50) by considering all combinations in terms of image quantity and quality. However, 3D SfM-based reconstructions did not allow us to accurately highlight their differences along the vertical axis of each species, with particular reference to the top of the stem (*AD*) for maize, tomato and olive tree (average rRMSE = 30.14%, 60.20% and 41.81%, respectively). Indeed, removing the *AD* significantly increased the average accuracy in basal (*BD*) and half-plant (*HD*) stem reproduction for maize (rRMSE = 25.86%), tomato (rRMSE = 15.44%) and olive tree (rRMSE = 28.78%). Consequently, 45 images with low resolution may be considered the best-fitted combination for measuring the diameters at different heights in tomato (R^2^ = 0.75, rRMSE = 19.73% and AIC = 19.11), while the highest input quantity (90) and quality are required for an inadequate reconstruction in maize and olive tree. 

#### 3.2.3. Petiole/Branch Inclination

The 3D models faithfully reproduced inclination of tomato petioles (*BI*; 0.66 ≤ R^2^ ≤ 0.95, 11.19% ≤ rRMSE ≤ 29.33% and 104.55 ≤ AIC ≤ 135.67) and olive tree branches (0.50 ≤ R^2^ ≤ 0.91, 6.09% ≤ rRMSE ≤ 19.56% and 34.44 ≤ AIC ≤ 53.56). As underlined by the AIC trend, higher quality of images has no significant effect on the goodness-of-fit of the simulated tomato petioles’ inclinations, while it significantly decreases the risk of underfitting or overfitting in olive tree. For this reason, the most effective and time-saving input combination for *BI* reconstruction of tomato and olive tree may be obtained by acquiring 30 images with medium (R^2^ = 0.85, rRMSE = 17.23% and AIC = 123.06) and high resolutions (R^2^ = 0.76, rRMSE = 16.16% and AIC = 45.81), respectively. 

#### 3.2.4. Leaf Inclination

A good agreement was found between observed and simulated *LI* in all combinations of images quantity and quality (R^2^ ≥ 0.77), except for maize reconstruction when using 30 medium-resolution images (R^2^ = 0.31). These good performances were associated with a very low rRMSE ranging from 0.58% to 9.85% for maize, 4.84% to 12.87% for tomato and 1.39% to 7.21% for olive tree. Hence, 30 high-resolution images represent the appropriate combination to correctly estimate *LI* of maize (R^2^ = 0.81, rRMSE = 5.93% and AIC = 66.65) and tomato (R^2^ = 0.98, rRMSE = 6.26% and AIC = 64.31), while the same quantity of images with medium quality may be sufficient for olive tree (R^2^ = 0.95, rRMSE = 5.78% and AIC = 18.79).

#### 3.2.5. Leaf Area

Three-dimensional (3D) reconstruction of *LA* showed the highest agreement and accuracy in maize (0.87 ≤ R^2^ ≤ 0.98 and 6.86% ≤ rRMSE ≤ 17.52%), while lower performances were obtained in tomato (0.34 ≤ R^2^ ≤ 0.79 and 11.01% ≤ rRMSE ≤ 19.69%) and olive tree (0.26 ≤ R^2^ ≤ 0.67 and 13.06% ≤ rRMSE ≤ 31.69%). The goodness-of-fit index has an antithetical trend between maize (33.55 ≤ AIC ≤ 58.97) on one side, and tomato (27.43 ≤ AIC ≤ 43.20) and olive tree (33.76 ≤ AIC ≤ 56.39) on the other. In particular, the highest fits for maize were observed when the number and quality of the images increased (Figure 5), while the risk of underfitting or overfitting in tomato and olive tree decreased with a lower quantity and resolution of input. As a result, the most efficient combinations for *LA* estimation may be considered 45 images with low quality in maize (R^2^ = 0.97, rRMSE = 6.86% and AIC = 39.74) and olive tree (R^2^ = 0.58, rRMSE = 16.38% and AIC = 41.64), and 30 images with low quality in tomato (R^2^ = 0.79, rRMSE = 11.01% and AIC = 27.43).

## 4. Discussion

The low-cost and automatic phenotyping platform proposed in this study has proven to be a useful tool for a reliable and affordable image-based phenotyping analysis on crops with extremely different canopy architectures. Within this pipeline, the image scanning strategy joined to the SfM-based 3D reconstruction technique resulted in an appropriate approach for the faithful estimation of the main plant phenotypic traits.

Indeed, differently from SfM imagery analysis based on a manual acquisition system [25,54], our platform is able to automatically acquire images and, thus, gather a pre-defined number of overlapped images, avoiding human mistakes (i.e., images out of focus and lack of scene features) [55]. Although the SfM methodology has been widely adopted for low-cost and accurate 3D plant modelling [33,34], only a few studies have focused on optimization of the reconstruction process in relation to different canopy architectures, even if they have been mainly fed with a fixed number of images of the same quality [40,56]. However, this approach prevented investigating the best input combination for an efficient modelling of each species, which is an essential reference for a practical application of SfM in agronomic and breeding programs. 

In this way, our study shows the capability of SfM in retrieving the architecture for herbaceous (i.e., maize and tomato) and tree crops (i.e., olive tree), establishing a compromise between measurements error and processing time. Indeed, although 90 images with the highest spatial resolution maximized the agreement and accuracy in estimating the total amount of morphometric traits for all the species, this combination required the longest processing time. Hence, in order to optimize the 3D reconstruction process, two main factors must be taken into consideration: the minimum number of images and the lowest spatial resolution that guarantee a correct extraction of the main morphological traits in a reasonable time. 

The performances related to each combination of quantity and quality of images are not easily generalizable but rather depend on specific parameters. In this context, a low number of images with poor spatial resolution negatively affected the accuracy of the 3D reconstruction, leading to higher errors in smaller objects (i.e., stem diameters and leaf area) [57]. In particular, the lower the ratio between the target size and the pixel, the higher the estimation error of the parameter [58]. As a consequence, images at higher resolution were required to reconstruct small traits (e.g., petioles inclination), due to the larger number of points within [36]. Conversely, results obtained on plant heights provided evidence that lower quantity and quality of images may be effectively merged for reconstructing large plant targets. 

The comparison between species with extremely different architectures highlights the need for a high-resolution scanning from multiple perspectives to retrieve complex canopies (e.g., olive tree), where a portion of inner structures are hidden by overlapping leaves [59]. This issue is more pronounced when acquiring images by hand-held cameras, as demonstrated in previous studies [60,61] where laborious graphical editors were needed to artificially complete the partially modeled branches. 

Coherently with the geometric complexity of the target-objects, a higher level of image resolution was required for crops with a curling-down leaf conformation (maize and tomato) [35,36], while a lower quality was necessary for flat leaves of olive tree. A similar result was obtained by Bernotas et al. [62], where the natural convex shape of *Arabidopsis* leaves led to poor accuracy in inclination extraction compared to flat surfaces. This could represent a limitation on the assessment of geometrical alterations in canopy structure when applied in other conditions, such as on stressed plants [63].

However, some technical aspects need to be improved to resolve the uncertainties in the estimation of traits (i.e., stem diameters and leaf area) when increases in images’ quantity and quality are not enough. In this context, low distances between sensor and plant [56] coupled with an increase in height and inclination of the camera, could be a cost-effective solution for reducing errors in canopy disclosure, keeping the potential of the zenith-point-of-view image acquisition system in vertical measurements unchanged. Nevertheless, errors can also be attributable to a manual and non-automatic extraction of the traits. For that reason, a fully automated point cloud segmentation could be useful for a more time-saving and accurate reconstruction of herbaceous and arboreal architectures. 

Looking at these considerations, we have demonstrated that specific combinations of quantity and quality of images are necessary for an accurate and efficient reproduction of specific objects with different size and shape. In particular, the input requirements we selected for maize and tomato models increase from *PH* and *BH* (30 L) to *LA* (45 and 30 L, respectively), *BI* (30 M) and *LI* (30 H), while olive tree reconstructions necessitated 30 (*PH*, *BH* and *LI*) and 45 (*LA*) images with low quality, or 30 high-resolution images (*BI*). Instead, we have not found any input combination that could guarantee a reproduction of *D* in a reasonable time, except for tomato (45 L).

Consequently, the use of 30 high-quality images (at maximum 4.88 µm/pixel spatial resolution) for reproducing the whole shape of both herbaceous (i.e., maize and tomato) and olive tree plants is a good compromise between a reliable reconstruction of the main morphometric traits (R^2^ = 0.72, rRMSE = 11.92% and AIC = 42.59) and processing time (Figure 4). 

Obviously, the suggested combinations may vary according to the plant growth and this issue should be taken into account when the proposed methodology is aimed at a practical application, such as crop monitoring. As a matter of fact, an increase in canopy density could generate overlapping surfaces and require a larger number of images with higher resolution for an accurate modelling. Instead, lower input requirements may be sufficient for the extraction of more developed organs due to their larger shape.

## 5. Conclusions

This study evaluated the performances related to different combinations of quantity and quality of images for a SfM-based 3D reconstruction of multi-species potted plants using a low-cost and automatic phenotyping platform equipped with a conventional photographic camera. The results provided evidence of the platform’s usefulness in representing the geometrical plant structure of each species considered, obtaining a reasonable compromise between time-processing and accuracy. Based on the case study, 30 images at 4.88 µm/pixel resolution resulted in the best input combination to efficiently reconstruct the whole shape of both herbaceous (i.e., maize and tomato) and tree plants (i.e., olive tree). However, we detected more time-saving strategies in terms of image quantity and quality to be applied when the use of the platform is aimed at the extraction of single parameters in herbaceous and tree crops. Solely the reconstruction of maize and olive tree stem diameters required an unrealistic processing-time for a practical application, whereas some improvements are needed for leaf area estimation at a higher level of detail. Accordingly, future efforts should focus on testing alternative image acquisition strategies, such as increasing the height and angle of the camera’s viewpoints and/or combining different sensors (e.g., Visible-Near Infrared spectroscopy). Moreover, the promising approximations in more complex traits’ extraction obtained in this study suggest that further 3D phenotyping tests should be conducted by processing between 45 and 90 images.

Nevertheless, the development of algorithms for automatic plant segmentation and morphometric traits’ extraction could effectively represent a time-saving solution in the future. On these bases, the platform might be useful for a low-cost and fast phenotyping of several plants simultaneously, regardless of the species considered. The promising results obtained in this study for a 3D reproduction of plant parameters highlighted that our platform represents an ideal system for further phenotyping applications. Indeed, the reliable 3D plant reconstruction and accurate estimation of vegetative parameters (i.e., plant height, leaf inclination and leaf angle) may be used for identifying the onset of biotic and abiotic stresses which are proxies for limitations in plant growth and production. In particular, upgrades of the current setup with sensors’ implementation may improve the low-cost and high-precision comprehensive analysis of plant responses to exogenous perturbations at the single-organ scale.

## Figures and Tables

**Table 1:** Statistical tests (R^2^ and rRMSE) and goodness-of-fit (AIC) for plant height (*HP*), branches height (*BH*), merged basal, half-plant and apical stem diameters (*D*), petioles/branches inclination (*BI*), leaf inclination (*LI*) and leaf area (*LA*) extracted from maize, tomato and olive tree 3D models using 90 images at 4.88 µm/pixel resolution.

*Trait*	Crop	R^2^	rRMSE	AIC
*HP*	Maize	0.99	5.03%	−2.74
*HP*	Tomato	0.99	5.69%	10.00
*HP*	Olive	0.83	1.86%	14.12
*BH*	Maize	0.97	8.37%	12.39
*BH*	Tomato	0.97	7.74%	52.30
*BH*	Olive	0.99	2.15%	88.04
*D*	Maize	0.23	11.80%	7.35
*D*	Tomato	0.67	21.86%	22.51
*D*	Olive	0.56	16.39%	35.61
*BI*	Tomato	0.95	11.19%	104.55
*BI*	Olive	0.91	6.09%	35.44
*LI*	Maize	0.99	0.58%	13.17
*LI*	Tomato	0.96	6.26%	63.58
*LI*	Olive	0.99	1.39%	12.38
*LA*	Maize	0.97	6.89%	40.22
*LA*	Tomato	0.36	19.69%	42.95
*LA*	Olive	0.50	28.37%	56.39

## Supplementary Materials

The following are available online at https://www.mdpi.com/1424-8220/20/11/3150/s1: Table S1: purchase prices (in € and $) of single-components used for the realization of (**a**) engine control module (including platform’s main structure and 10 rotating plates), (**b**) data logger module and (**c**) data acquisition module. The EUR–USD exchange was made with reference to the conversion rate of 07/05/2020.
